# Sugar Signaling During Fruit Ripening

**DOI:** 10.3389/fpls.2020.564917

**Published:** 2020-08-28

**Authors:** Sara Durán-Soria, Delphine M. Pott, Sonia Osorio, José G. Vallarino

**Affiliations:** Departamento de Biología Molecular y Bioquímica, Instituto de Hortofruticultura Subtropical y Mediterránea “La Mayora”, Universidad de Málaga – Consejo Superior de Investigaciones Científicas (IHSM-UMA-CSIC), Málaga, Spain

**Keywords:** sucrose, sugars, signaling, ripening, hormones, pigments, stress, fruit

## Abstract

Sugars play a key role in fruit quality, as they directly influence taste, and thus consumer acceptance. Carbohydrates are the main resources needed by the plant for carbon and energy supply and have been suggested to be involved in all the important developmental processes, including embryogenesis, seed germination, stress responses, and vegetative and reproductive growth. Recently, considerable progresses have been made in understanding regulation of fruit ripening mechanisms, based on the role of ethylene, auxins, abscisic acid, gibberellins, or jasmonic acid, in both climacteric and non-climacteric fruits. However, the role of sugar and its associated molecular network with hormones in the control of fruit development and ripening is still poorly understood. In this review, we focus on sugar signaling mechanisms described up to date in fruits, describing their involvement in ripening-associated processes, such as pigments accumulation, and their association with hormone transduction pathways, as well as their role in stress-related responses.

## Introduction

Sugars are the main structural components of plant tissues, providing energy and carbon building blocks for growth and reproduction. In particular, fruit and seed development, crucial processes for global crop production, depends on the transport of photoassimilates through the phloem and assimilation from source to sink tissues (Ruan et al., 2012; Liu et al., 2013). Numerous studies, including metabolomic-driven approaches, have reported sugar content behavior during fruit growth and ripening, as soluble sugar accumulation determines fruit sweetness at harvest, an essential parameter of fruit quality (Deluc et al., 2007; Lombardo et al., 2011; Osorio et al., 2012; Vallarino et al., 2018). Furthermore, several studies pointed out that both sucrose and the main hexoses (glucose and fructose) originated from sucrose degradation, are involved in signaling and regulation of plant development (Ruan, 2012; Xu et al., 2012; Tognetti et al., 2013). Indeed, sugars may control in a direct or indirect way several processes, including photosynthesis, nitrogen uptake, defense mechanism, hormone balance, or secondary metabolism (Smeekens et al., 2010; Lecourieux et al., 2014). This control is mainly exerted by regulating gene expression, affecting mRNA stability or protein translation/stability (Koch, 1996; Wiese et al., 2004). Plants have developed mechanisms by which they perceive sugar fluxes, known as sugar sensing, and by which they control sugar-mediated responses, allowing them to adapt their activity at the cellular level based on sugar status and maintain homeostasis (Lecourieux et al., 2014).

### Sugar Metabolism in the Fruit

Sucrose is the major fixed carbon (C) form of carbohydrates for long-distance transport through the phloem from leaves sources to non-photosynthetic sink organs, including developing fruits (Julius et al., 2017). Once sucrose reaches the sink cells, sucrose is hydrolyzed by sucrose synthases or invertases into fructose and glucose, which help maintaining sink strength. Sucrose synthases are mainly involved in the synthesis of carbohydrate polymers, i.e. starch or cellulose, or in the generation of energy, necessary for the production of a myriad of compounds which help fruit development and seed dispersal (Braun et al., 2014).

Invertases are classified into three groups based on their subcellular localization: cytoplasmic invertases (CIN), vacuolar invertases (VIN), and cell wall invertases (CWIN) (Li et al., 2012). Invertases play a regulatory role in plant growth and development, and VIN and CWIN are also involved in biotic and abiotic stress responses, connecting sucrose and hexose signaling with stress adaptation (Li et al., 2012; Li et al., 2016). Apart from their roles in stress responses, which evidences in fruits will be discussed further in this review, invertases and sucrose synthases are key factors determining the growth of bulky sinks (Xu et al., 2012). As an example, *LIN5* gene, which encodes a CWIN in tomato, is considered one of the main enzymes affecting sugar uptake into the fruit (Fridman et al., 2004; Baxter et al., 2005; Zanor et al., 2009; Vallarino et al., 2017). In addition, CWIN activity increases during ovary-to-fruit transition, possibly to facility phloem unloading and to produce a glucose signal, which positively regulate cell division and thus, fruit set (Palmer et al., 2015).

### Sugar Transporters

Sugar transport is highly regulated and several transporters are involved in sucrose export from photosynthetic cells, phloem loading and unloading. Additionally, once in the fruits, part of the sucrose-derived hexose pool is also transported to the vacuole as a strategy to maintain sink strength (Lemoine et al., 2013; Liu et al., 2013; Lecourieux et al., 2014). These transporters can be divided into three main families: sucrose (SUT), monosaccharide (MST), and sugar will eventually be exported (SWEET) transporters (Julius et al., 2017; Jeena et al., 2019).

Both SWEET and SUT proteins are involved in phloem loading; while SWEET transporters act as facilitated diffusers, transporting sugars across membrane down a concentration gradient (Chen et al., 2010; Chen et al., 2012). SUTs are sucrose/proton symporters, using stored energy to drive active sucrose movement (Gaxiola et al., 2007). Phloem unloading in developing fruits can occur apoplasmically into cell wall matrix, as described in apples, or symplasmically through plasmodesmata in recipient cells (Wang and Ruan, 2013; Braun et al., 2014). In some fruits, such as tomato, both apoplasmic and symplasmic unloading take place and is under developmental control (Braun et al., 2014). In this process, sucrose effluxers, such as SWEET or SUT proteins, are involved in export sucrose from phloem, and sucrose or hexose influxers uptake sugars to the recipient sink cells. In apoplasmic unloading, the co-expression of cell wall invertases, which convert sucrose to hexoses, with MST generally occurs (Ruan et al., 1997; Jin et al., 2009). Once in the cell, other transporters belonging to the MST, SUT, and SWEET families, carry sucrose, fructose, and glucose into the vacuole, where most sugars are stored (Wormit et al., 2006; Aluri and Büttner, 2007; Cheng et al., 2018). In particular, members from the MST family, namely tonoplast sugar/monosaccharide transporters (TST/TMT), have been described to be involved in this process in high-sugar storage organs, such as sugar beet roots or melon and watermelon fruits (Hedrich et al., 2015; Cheng et al., 2018; Ren et al., 2018). *Arabidopsis* genome contains three isoforms of *TMT*; it was reported that *TMT1* expression increased during grape berry development (Terrier et al., 2005), and the study of Afoufa-Bastien et al. (2010) suggests that both TMT1 and TMT2 could play a role during fruit ripening. A recent study reported that the overexpression of watermelon *ClTST2* is positively correlated with sugar accumulation during fruit ripening in watermelon, and its regulation by a sugar-induced transcription factor (TF) will be discussed in another section of this review (Ren et al., 2018).

### Sugar Signaling in the Fruit

In most species, sucrose is the main form of long-distance transported sugars through the plant; additionally, it can also initiate signaling pathways and affect gene expression, even if a specific receptor has not been found yet. The ability of sugars as regulators of gene expression has been long known, and glucose has been reported to regulate cell division (Smeekens et al., 2010; Wang et al., 2014; Palmer et al., 2015), while sucrose is involved in the accumulation of reserves during embryogenesis (Borisjuk et al., 2002; Borisjuk et al., 2003). Sucrose participation in signaling has been long known as demonstrated by Maas et al. (1990), who described how it can inhibit maize *Shrunken* gene promotor or by Wenzler et al. (1989) and Jefferson et al. (1990), which showed that *patatin-1* promoter was specifically induced by sucrose in potato tubers. More recently, Wiese et al. (2004) observed that sucrose induces the repression of a basic region leucine zipper (*AtbZIP*) transcription factor (TF) in *Arabidopsis*, while other sugars such as glucose and fructose have no effect (Kanayama, 2017). Additionally, other bZIP-sucrose sensitive TFs have been discovered, such as *CAREB1* in carrot, which is sensitive to both sucrose and abscisic acid (ABA) (Guan et al., 2009; Wind et al., 2010). Regarding fruit, it was verified that hexose transporter genes involved in sugar metabolism, were induced by sucrose, and that their promotor contains sucrose-responsive motifs or “sucrose box” (Çakir et al., 2003; Jia et al., 2016). For instance, *VvHT1* gene, which encodes a monosaccharide transporter in grape, contains two sugar boxes and is regulated by several sugars, including sucrose, glucose, and palatinose (Atanassova et al., 2003).

Furthermore, sugar and hormones crosstalk has already been described in plant development (Eveland and Jackson, 2012). However, most studies have focused on seeds and early plant development, and very few on sugar signaling role during fruit ripening. It is more recently that sugars have been associated with key processes related to fruit ripening, such as pigment accumulation, and their regulating roles in this complex process, alone or in a crosstalk with hormone signaling (Jia et al., 2013; Liu et al., 2013; Li et al., 2016), are discussed below.

## Sugar Signaling in Fruit Development and Ripening and Its Crosstalk With Hormones

### Sucrose

Several studies have confirmed that sugar (and especially sucrose) metabolism is a key process in fruit development, promoting both its own accumulation and driving ripening events (Ruan et al., 2012; Tognetti et al., 2013). Indeed, a high correlation between endogenous sucrose and ripening phase changes has been observed in orange fruits (Wu et al., 2014). Similarly, late ripening has been observed when the concentration of sucrose in orange spontaneous mutant decreased (Zhang Y. J. et al., 2014). Exogenous sucrose treatment also improves strawberry ripening, accelerating the coloration of the fruit and allowing it to reach the ripe stage earlier (Jia et al., 2011; Jia et al., 2013; Luo et al., 2019). As well, the effect of exogenous sucrose in harvested tomatoes at the green mature stage by accelerating ripening and postharvest processes in the fruits has been described (Li et al., 2016).

Sucrose controls genes involved in sugar metabolism, such as the expression of starch biosynthetic enzymes, starch synthase and β-amylase (Nakamura et al., 1991; Wang et al., 2001). The investigation of Li et al. (2002) showed that in tomato fruit sucrose induced the expression of *ApL3*, which encodes an ADP-glucose phosphorylase, a key regulatory enzyme in starch biosynthesis. *CIN* genes, which are implicated in sucrose degradation and have a fundamental role in the maintenance of sugar homeostasis in the cytoplasm, are also regulated by sucrose itself, as demonstrated in tomato fruits (Ruan et al., 2010; Li et al., 2016). Furthermore, exogenous sucrose treatment in tomato fruits resulted in the downregulation of an invertase inhibitor gene (*INVINH1*). *CIN6*, unlike most sucrose breakdown genes, is downregulated when sucrose increases (Li et al., 2016). Interestingly, increased sucrose degradation by overexpressing *CIN* or *LIN5* genes did not enhance glucose and fructose levels, and seems to confirm that sucrose acts, at least partially, independently of hexose-derived signals in certain signaling pathways (Albacete et al., 2014; Li et al., 2016). Another proof of sucrose regulator role during ripening is its interaction in tomato with the MADS-box TF *RIN*. *RIN* is a global regulator of fruit ripening, well studied in this fruit (Ng and Yanofsky, 2001; Vrebalov et al., 2002; Giovannoni, 2007; Osorio et al., 2012); interestingly, Qin et al. (2016) observed that several genes involved in sugar metabolism, were differentially expressed in the *rin* mutant compared to Aisla Craig tomato wild type fruits. In particular, lower VIN expression was observed in the *RIN*-silenced fruits, while a VIN inhibitor (*SlVIF*) showed higher expression. Interestingly, in *SlVIF*-silenced lines, *RIN* expression was affected and the green to red transition of the fruits was retarded in comparison with wild-type and *SlVIF*-overexpressed lines (Qin et al., 2016). Concomitant with the impact on color change, *SlVIF* silencing results in diminished ethylene production, and in the alteration of transcript/protein profiles involved in fruit maturation, suggesting a key role of this invertase inhibitor in controlling tomato ripening (Qin et al., 2016).

Sucrose transport, from leaves sources to sink organs is more important than *de novo* synthesis in the fruit (Hackel et al., 2006; Jia et al., 2013). Thus, regulation of SUT transporters is an important factor which demonstrates the role of endogenous sucrose in fruit development and ripening. In this way, *SUT1* silencing clearly reduces sucrose concentration in the fruit, delaying strawberry and citrus ripening (Jia et al., 2013; Wu et al., 2014). In apple, cytochrome B5 has been proposed as a possible sucrose sensor, since it interacts with MdSUT1 and MdSOT6, a sorbitol transporter (Fan et al., 2009). Another suggested sucrose sensor is SUT2, which may couple sucrose sensing with G protein and thus mediate sugar regulation on the expression of genes involved in fruit development (Barker et al., 2000; Wind et al., 2010). Alternatively, sucrose could be transformed into glucose and fructose, and these sugars would be responsible to regulate gene expression (Rolland et al., 2006; Zhang Y. J. et al., 2014). However, it was recently demonstrated that the downregulation of the genes involved in the catalysis of sucrose into fructose and glucose, *i.e.* α-glucosidase and invertase, did not affect the expression of *SUT2* (Luo et al., 2019). Furthermore, no significant differences were observed in the expression of *LeSUT1*, *LeSUT2*, and *LeSUT4* when sucrose (25 µl, 500 mM) were injected into the pedicle of tomato fruit recently harvested (Li et al., 2016). Hexokinase (HXK), a very important enzyme for monosaccharide metabolism, has also been proposed as a possible sugar receptor. Indeed, HXK1 can form a complex with a vacuolar H^+^-ATPase and the 19S subunit of the proteasome, that directly modulates the expression of target genes (Cho et al., 2006). While most SUT transporters are localized in plasma membrane, some members of SUTII subfamily were found associated with vacuole membrane and their overexpression results in increased sucrose accumulation in this organelle (Ma et al., 2017; Ma et al., 2019; Peng et al., 2020). Surprisingly, a negative correlation between apple tonoplast *MdSut4.1* expression and sucrose accumulation was observed, and was confirmed by transient overexpression in strawberry fruit, suggesting a function in sucrose remobilization out of the vacuole (Schneider et al., 2012; Peng et al., 2020). Taken together, these observations could propose MdSUT4.1 as a key component of the regulation of apple fruit sugar accumulation.

Recently, the study of Ren et al. (2018) shows that watermelon genome contains three *TST* orthologs, being *ClTST2* the principal sugar transporter into vacuoles. *SUSIWM1*, a sugar-induced TF, binds to a SURE element, located in a region of *ClTST2* promoter, and induces *ClTST2* expression stimulating sugar loading and storage into vacuoles of watermelon fruit cells (Ren et al., 2018). Interestingly, SURE elements were described in potato to be localized in promoters of genes regulated by sucrose (Grierson et al., 1994), suggesting that sugar storage in vacuoles during fruit ripening, mediated by TST2 transporters, is controlled by sucrose itself. Three *TST* genes were also found in melon genome, and *CmTST2* presents the highest correlation with sugar accumulation during fruit ripening (Cheng et al., 2018). Stable overexpression of *CmTST2* in melon and cucumber plants allowed to establish the role of this transporter in sugar accumulation during fruit ripening, as ripe overexpressed fruits contained higher levels of fructose, glucose, and sucrose, compared to control fruits (Cheng et al., 2018). Moreover, *TST2* gene was transiently overexpressed in strawberry fruit and, as a result, the fruits presented a lower concentration of cytosolic sugar, due to an increased rate in sucrose introduction into the vacuole. More interestingly, strawberry ripening was delayed in the transient-overexpressed fruits, compared to the control, possibly as the consequence of sugar signaling involvement in this physiological process (Cheng et al., 2018). *CmTST1* expression is very high during the early stage of melon fruit development, and then decreased rapidly, concomitant with glucose and fructose accumulation pattern in this fruit, suggesting a role of this ortholog in hexose storage in the vacuole. It is worth noting that sugar-responsive elements were identified in the promotor region of the three *CmTST* genes (Cheng et al., 2018). In this sense, the functions and characteristics of tonoplast transporters in fleshy fruits and their involvement in ripening need more research.

Sucrose also regulates anthocyanin and carotenoid biosynthesis, two groups of secondary metabolites involved in fruit coloration, and will be detailed in other sections of this review. Experiments have recently been conducted to demonstrate that the role of sucrose in ripening is not due to its degradation into fructose and glucose. Indeed, Jia et al. (2013, 2017) demonstrated that turanose, a non-metabolizable sucrose analogue, has the same effect than sucrose on the expression of ripening genes in grape and strawberry.

### Trehalose-6-Phosphate: Its Dual Function in Sucrose Sensing and Its Regulation

In addition to the aforementioned main sugars present in the fruit, ***i.e.*** fructose, glucose, and sucrose, some minor sugars, such as trehalose-6-phosphate, an intermediate in the disaccharide trehalose metabolism, have been proposed as a signal for carbon availability and homeostasis, and could play an essential role in both plant development and abiotic stress tolerance (Yadav et al., 2014; Kretzschmar et al., 2015; Nuccio et al., 2015). In particular, it was demonstrated that trehalose-6-phosphate levels in different plant tissues was positively correlated with sucrose content, and that the addition of sucrose or sugars which can be metabolized to this disaccharide, was able to induce a fast rise in trehalose-6-phosphate content, suggesting that the latter is a specific signal monitoring sucrose status (Debast et al., 2011; Martínez-Barajas et al., 2011; Nunes et al., 2013; Yadav et al., 2014; Zhang et al., 2015; Figueroa and Lunn, 2016). Furthermore, as the correlation between sucrose and trehalose-6-phosphate was conserved in *Arabidopsis* plants which constitutively expressed the *E. coli* ***trehalose-6-phosphate synthase*** or ***trehalose-6-phosphate phosphatase***, involved in trehalose-6-phosphate synthesis and catabolism, respectively, it could be concluded that trehalose-6-phosphate is both a signal of sucrose availability and a negative feedback regulator of sucrose content, maintaining it in a closely controlled range (Yadav et al., 2014; Figueroa and Lunn, 2016).

Trehalose-6-phosphate is thought to act through the action of the sucrose non-fermenting-related kinase-1 (SnRK1) pathway, even if the exact molecular mechanism by which they interact is still imprecise (Delatte et al., 2011; Ponnu et al., 2011; Figueroa and Lunn, 2016). SnRKs are kinase proteins involved in signal transduction of carbon and nitrogen, being key regulators and sensors in plant carbohydrate metabolism (Emanuelle et al., 2016). Regarding fruit set and development, a study in cucumber suggests that trehalose-6-phosphate and SnRK1-mediated pathways are involved in “first-fruit inhibition,” a phenomenon in which first fruit set has an inhibitory effect on the growth of subsequent fruits (Zhang et al., 2015). Indeed, high trehalose-6-phosphate levels and low SnRK1 activity in the peduncle of the first fruit may participate in increasing sink strength, by modulating the expression of *CsAGA1*, an alpha-galactosidase, which catalyzes the hydrolysis of stachyose, the main carbohydrate transport form in cucumber, to sucrose (Zhang et al., 2015).

Wang et al. (2012) was the first study to show that the heterologous overexpression of *SnRK1* from *Malus hupehensis* in Sy12f tomato can promote fruit ripening by increasing both starch and soluble sugars. In addition, the fruits of *MhSnRK1* overexpressed plants displayed early ripening. Furthermore, these fruits showed greater diameter. Increased carbon metabolism in the leaves could explain the higher available carbon in the fruits stored as starch and soluble sugars (Wang et al., 2012). A second study overexpressing *Prunus* *persica* SNF1-related kinase α subunit (*PpSnRK1α*) in Sy12f tomato allowed to go deeper in understanding the molecular mechanism by which SnRK1 regulates fruit ripening (Yu et al., 2018). By yeast-two-hybrid experiment and BiFC assay, positive interaction was detected between PpSnRK1α and RIN. Furthermore, in *PpSnRK1α* overexpressing fruits, *RIN* expression, together with the levels of other TFs directly controlled by RIN, were enhanced, suggesting a regulation by SnRK1 both at the post- and transcriptional level (Yu et al., 2018).

*FaSnRK1α* overexpression and silencing in Miaoxiang 7 strawberry confirmed its importance in fruit ripening, by accelerating and delaying it, respectively. In addition, FaSnRK1α was seen to increase sucrose content in the fruit, by controlling the expression of genes involved in its transport and metabolism (Luo J. et al., 2020). The physical interaction between FaSnRK1α and several enzymes involved in sucrose hydrolysis and synthesis, including the sucrose phosphate synthase FaSPS3, which has been directly associated with sucrose accumulation during strawberry fruit ripening, was also demonstrated by yeast two-hybrid (Wei et al., 2018; Luo J. et al., 2020).

The relation between trehalose-6-phosphate and SnRK1 protein seems to be conserved in species that transport other sugars than sucrose. Kiwifruits transport both sucrose and the trisaccharide planteose through the phloem to heterotrophic tissues (Boldingh et al., 2015). Under carbon starvation, the low levels of trehalose-6-phosphate in Zes006 kiwifruit removed the inhibition on SnRK1, which initiates the signaling of low-energy status (Delatte et al., 2011; Nardozza et al., 2020).

Even if trehalose-6-phosphate acts as a signal and regulator of sucrose levels in plants, the nexus between both sugars is still not so well understood, and a possible role in fruit ripening has not yet been clearly established. For example, a study in Cabernet Sauvignon grape during berry development did not observe a clear correlation between sucrose or hexoses and trehalose-6-phosphate levels (Dai et al., 2013). A possible explanation for this lack of direct correlation could be the accumulation in different organelles—vacuole for sugars, cytosol for trehalose-6-phosphate. However, by building a metabolite-metabolite correlation network, Dai et al. (2013) demonstrated that trehalose-6-phosphate was the most connected compound in the network, suggesting its participation in multiple metabolic pathways. Interestingly, a strong and positive correlation was observed between trehalose-6-phosphate and sorbitol content during Gala apple fruit development (Zhang W. et al., 2017). Sorbitol, instead of sucrose, is the main transported sugar from leaves to sink tissues in some economically important crop species belonging to the Rosaceae family, such as apples or pears, and is mainly found in the cytosol of the sink cells (Rennie and Turgeon, 2009; Shangguan et al., 2014). Furthermore, trehalose-6-phosphate levels are negatively correlated with sucrose and other sugars during apple fruit ripening, and positively correlated with sucrose in sorbitol-limited fruits (obtained by thinning), indicating that sucrose may be regulated by a specific pathway which acts only under abnormal growth situation, such as carbon starvation (Zhang W. et al., 2017). In addition, sorbitol:trehalose-6-phosphate ratio was strongly linked to starch content and correlated with glucose, one of the two main products of starch degradation, which occurs in the later stages of apple ripening (Zhang W. et al., 2017). Even if the relation between trehalose-6-phosphate and starch content needs further confirmation, it could nevertheless outline the possible role of this sugar in starch breakdown during fruit development, as previously described in potato tubers by Kolbe et al. (2005).

### UDP-Sugars

UDP-sugars are an activated form of sugars which act as donors for different biosynthetic reactions. Although little evidence exists at the present time, a possible role in plant signaling has been suggested based on studies in animals, which outlined a role for uridine-5’ disphosphate-glucose (UDPG) as an extracellular signaling molecule, that is sensed by G-protein-coupled receptors (Chambers et al., 2000; Harden et al., 2010; Lazarowski and Harden, 2015). Additionally, as sucrose, a known signaling molecule in plant, is rapidly split into glucose, UDPG, and fructose, a potential role of these breakdown products in sugar signaling cannot be ruled out (Hummel et al., 2009). Furthermore, the lack of identified sucrose receptor might imply a signaling function of its breakdown products (Janse van Rensburg and Van den Ende, 2018). However, as UDPG is directly related to the concentration of sucrose, and therefore, to the accumulation of glucose, fructose, and trehalose-6-phosphate, it cannot be ruled out that UDPG can cause an imbalance in the levels of other metabolites involved in signaling instead of being a signaling sugar itself. Deeper studies on the possible signaling role of UDPG seems nevertheless necessary, as UDPG function in plant growth and development is well described, and a recent study suggests that UDPG enhances biomass accumulation in sugarcane because of its rapid conversion to sucrose (Wai et al., 2017). UDPG pyrophosphorylase, an enzyme involved in UDPG formation and which is differentially expressed under stress conditions (Ciereszko et al., 2001; Meng et al., 2007), has been shown to be a regulator of programmed cell death (Janse van Rensburg and Van den Ende, 2018; Xiao et al., 2018). A very recent study has suggested that UDPG might have an important role in the induction of reactive oxygen species generation and lesion mimics in rice (Xiao et al., 2018), nevertheless, further studies are required to understand this process and the possible role of UDPG or any other UDP-sugar in plant signaling and fruit ripening.

### Sucrose and Abscisic Acid

The function of ethylene in climacteric fruit ripening has been long known, however, in non-climacteric fruits, ABA seems to play a more important role (Jia et al., 2016). Indeed, ABA increases during orange ripening and promotes fruit color formation (Rodrigo et al., 2006), while in strawberry, another non-climacteric fruit, ABA modulates growth and softening, as well as the synthesis of anthocyanins and volatiles compounds (Jia et al., 2011), whereas ethylene role is more limited (Chervin et al., 2004; Trainotti et al., 2005; Merchante et al., 2013; Jia et al., 2016). Furthermore, in early tomato development, a climacteric fruit, the peak of ABA precedes the peak of ethylene, suggesting that both hormones promote together fruit ripening (Li et al., 2016).

**Figure 1:**
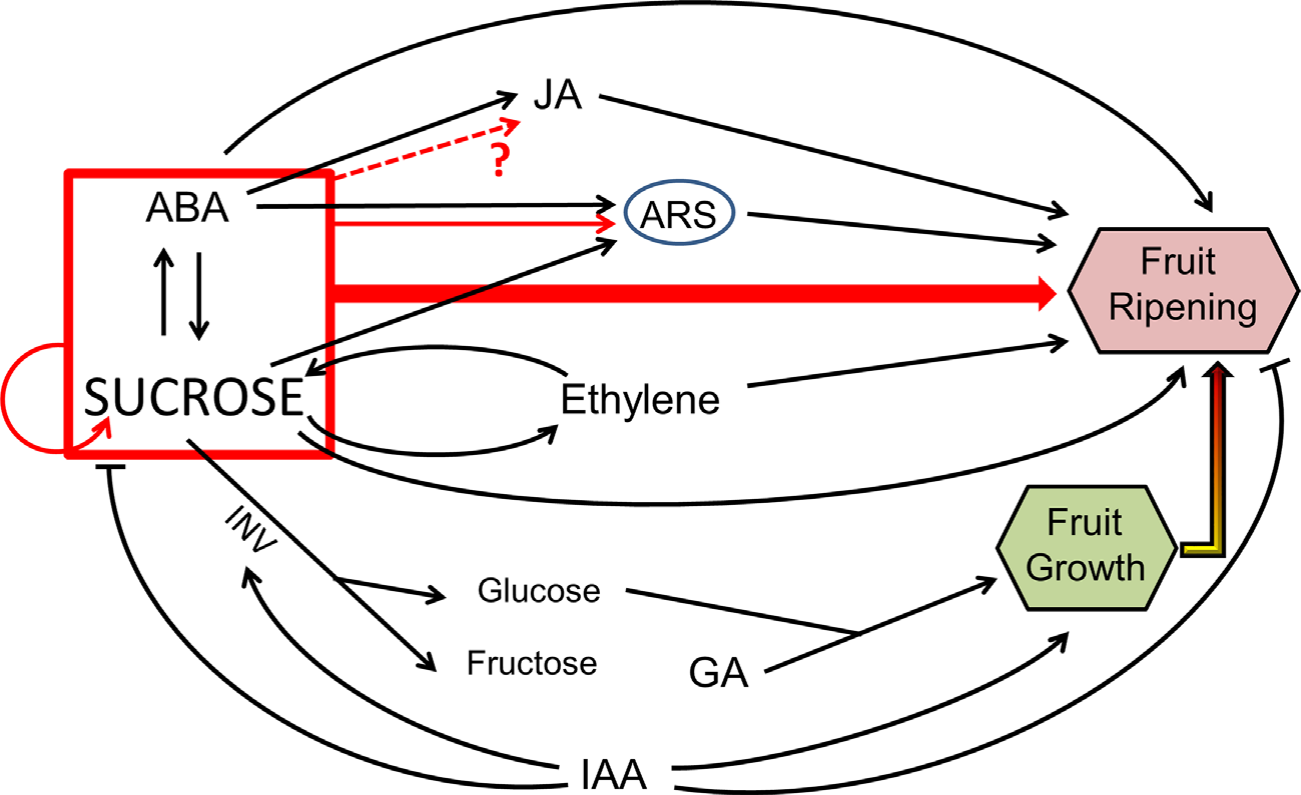
General schema of sugar signaling and its crosstalk with hormones. In the first stages of fruit development IAA and GA promote cell expansion and elongation, meanwhile IAA represses sucrose accumulation and stimulates its degradation by invertase (INV) into glucose and fructose, which are also necessary for fruit growth. During ripening, sucrose can activate several genes which enhanced ABA, ethylene, and JA levels, as well as ASR genes, thus promoting fruit ripening processes; in addition, sucrose by itself can promote fruit ripening too. Red lines symbolize a synergetic action between sucrose and ABA. This model is inspired in Jia et al., 2013; Wu et al., 2014; Zhang Y. J. et al., 2014; Zhang Y. et al., 2014; Jia et al., 2016, and Murayama et al., 2015.

An idea that is becoming more and more expanded is that sugar signaling and ABA are involved in a crosstalk which regulates fruit ripening, being this interaction especially evident in non-climacteric fruits (**Figure 1**; Gambetta et al., 2010; Cherian et al., 2014). A recent model in apple plant established the role of ABA in promoting soluble sugar accumulation, by activating the expression of genes involved in sugar transport, such as *MdSUT2*, and starch degradation. As a consequence, ABA signaling contributes greatly to enhance sweetness, a very important trait for fruit quality (Ma et al., 2017). Furthermore, other studies focused on the interaction between ABA and sucrose signaling in promoting ripening-associated processes. Indeed, it was recently described that treatments with exogenous ABA and sucrose greatly accelerate strawberry and grape fruits ripening, being the effect obtained much more intense than with either sucrose or ABA alone (Jia et al., 2016; Jia et al., 2017; Luo Y. et al., 2020). Grapes were collected and treated with different concentrations of ABA and sucrose (100 μM ABA and 16 mM sucrose) (Jia et al., 2017) and green-stage strawberries were sprayed with 95 μM ABA and 100 mM sucrose, and collected fully ripe (Luo Y. et al., 2020). Differentially expressed genes have been observed between the three aforementioned treatments (ABA, sucrose and ABA + sucrose), suggesting that the differences are due to the synergistic action between ABA and sucrose, and not due to the additive effects of two independent routes (Luo Y. et al., 2020). In particular, recent studies have reported that this interaction between ABA and sucrose exerts control on the synthesis of anthocyanins in strawberry fruit (Jia et al., 2016) and starch in maize (Huang et al., 2016). During peach ripening, a crucial connection was outlined between ABA and sucrose, admitting a possible crosstalk between the two signaling molecules (Falchi et al., 2013). Furthermore, other studies in citrus fruit suggest that sucrose could be a key regulatory molecule of ripening, together with the phytohormones ABA, ethylene, and jasmonic acid (JA) (Wu et al., 2014; Zhang Y. J. et al., 2014). Indeed, ABA, ethylene, and sucrose pathways were clearly different between a spontaneous late-ripening mutant and wild type orange, as shown by the significant differences in the levels of these three metabolites. However, there is still need to clarify which is the most important factor responsible for the delay in ripening observed in the spontaneous mutant (Wu et al., 2014).

In addition, sucrose and ABA could concurrently regulate the expression of some genes during fruit development, including genes involved in sugar metabolism and accumulation. For example, sucrose and ABA induce sucrose synthase activity in rice (Tang et al., 2009), although more studies are needed to understand the molecular mechanisms of this process. Another study in Cabernet Sauvignon grape focused on the effect of sugar status on the expression of genes in grape berry cell suspension. Following a transcriptomic approach, Lecourieux et al. (2010) observed that the expression of a *glycogen synthase kinase3*, *VvSK1*, was enhanced both by ABA and exogenous sucrose, underlining the intercommunication between sugars and hormone signaling pathways. The overexpression of *VvSK1* in cell suspension enhanced the expression of some MST and as a consequence, increased the levels of soluble sugars in the cell (Lecourieux et al., 2010). In grape berries, *VvSK1* expression increased after the veraison stage, concomitant with sugar and ABA levels, suggesting that sugar could regulate fruit sugar content by modulating VvSK1 action (Lecourieux et al., 2014).

*ASR* (ABA stress ripening) belongs to a gene family which encodes small basic proteins induced by ABA, abiotic stresses and during fruit ripening. The expression of *ASR* genes has been reported in several fruits, such as tomato (Iusem et al., 1993), pomelo (Canel et al., 1995), apricot (Mbeguie-A-Mbeguie et al., 1997), and grape (Çakir et al., 2003). The first evidence about ASR physiological function in sugar and ABA signaling pathways was found by Çakir et al. (2003). Indeed, they observed in grape cell suspension grown in medium supplemented with sucrose alone (58 mM) or with sucrose and ABA (10 µM) that expression of a grape *ASR* gene (*VvMSA*), which is upregulated during grape fruit ripening, was inducible by sucrose, and also this induction was enhanced in presence of ABA. It was later established that *ASR* was also involved in the crosstalk between ABA and sucrose during ripening, as its expression could also be induced by these hormone and sugar during tomato, peach, and strawberry fruit maturation (Chen et al., 2011; Jiaxing et al., 2018). *ASR* expression in tomato and strawberry pointed out a role in fruit ripening and was also induced by ABA and sucrose, in two different pathways, which can work independently or together (Jia et al., 2016). Indeed, sucrose and ABA inhibitor nordihydroguaiaretic acid treatment induced *ASR* expression, while nordihydroguaiaretic acid alone did not induce it, suggesting that sucrose can act separately from the hormone response (Jia et al., 2016).

Transient overexpression of peach *ASR* gene in tomato fruits resulted in changes in cell wall degradation and pigment synthesis, confirming ASR role in promoting fruit ripening, as observed in overexpression and silencing experiments in tomato and strawberry fruits (Jia et al., 2016; Jiaxing et al., 2018). In fact, Jia et al. (2016) suggested that *ASR* acts as a switch, controlling ripening-related genes in response to activation by ABA and/or sucrose. Furthermore, ASR may also act as a transcription factor, as suggested by Çakir et al. (2003), which showed that it could bind two sugar-responsive elements (sugar boxes) present in the promotor region of *VvHT1*, enhancing the expression of this hexose transporter. Peach *ASR* gene was also able to bind the unique *hexose transporter* gene present in this species, confirming its role as TF, acting in response to ABA and sucrose signaling, which cooperatively regulated its transcription (Jiaxing et al., 2018). *Hexose transporter* gene induction by *ASR* was also confirmed in tomato and strawberry, and, as expected, was enhanced by sucrose, ABA, or sucrose + ABA (Jia et al., 2016).

Besides the synergistic effect that ABA and sucrose shows during fruit ripening, it seems that sucrose is also involved in enhancing ABA levels (**Figure 1**). Jia et al. (2011, 2013) observed an increase in the level of ABA after glucose and sucrose treatments, accelerating strawberry fruit ripening, being sucrose effect more pronounced. As a consequence of this observation, this sugar has been proposed to induce the expression of key genes involved in ABA biosynthetic pathway, such as 9-cis-epoxycarotenid dioxygenase (*NCED*) and beta-glucosidase (*BG*) (Jia et al., 2013). A recent investigation confirmed that ABA (95 µM) and sucrose (100 µM) treatments resulted in the overexpression of *FaNCED1* and *FaNCED2* in sprayed strawberry fruit (Luo et al., 2019). Nevertheless, it is worth noting that in the absence of ABA, sucrose treatment had no effect on the expression of *FaNCED*, even if it was able to significantly upregulated the expression of *FaBG.* Taken together, these data suggest that sucrose level can modulate ABA content in strawberry fruit (Luo et al., 2019).

Silencing of the E1 component subunit alpha of the pyruvate dehydrogenase, involved in glucose metabolism and in the conversion of pyruvate to acetyl-CoA, has been reported to inhibit glycolysis, and accelerate ripening in strawberry, as a consequence of ATP and respiration repression (Wang et al., 2017). Luo Y. et al. (2020) suggested that this inhibition of glycolysis is an important factor for ripening and could be induced by ABA and sucrose, supported by the observation that *GAPDH*, another key enzyme in glycolysis, was downregulated after treatment with ABA and sucrose in strawberry fruit. Furthermore, they proposed that hydrogen peroxide (H_2_O_2_) could affect the synergetic action of ABA/sucrose showing a short inhibition of glycolysis during strawberry fruit ripening, as the levels of endogenous H_2_O_2_ was induced by ABA, sucrose, and ABA + sucrose treatments. Enhanced H_2_O_2_ level is an indication of oxidative stress, which may inactivate GAPDH, resulting in a shift of the carbon flux from glycolysis towards the pentose phosphate pathway to generate reducing equivalents (Krüger et al., 2011). Interestingly when strawberry fruits were treated with reduced glutathion, an important antioxidant, H_2_O_2_ content was decreased and an important delay in fruit coloration was also observed, confirming the role of H_2_O_2_ in connecting ABA, sucrose, and fruit ripening (Luo Y. et al., 2020).

### Sucrose and Other Hormones

Ethylene has long been known for its function in fruit ripening, being the key hormone controlling this process in climacteric fruits (Giovannoni, 2007). It has already been reported that ethylene promotes sucrose accumulation in several fruits (Chervin et al., 2006; Choudhury et al., 2009; Farcuh et al., 2018), but it has also been observed that sucrose can also stimulate ethylene biosynthesis, promoting the postharvest ripening of tomato and kiwifruits (**Figure 1**; Li et al., 2016; Fei et al., 2020). Sucrose was able to enhance the expression of ethylene receptor (*ETR*) genes, *SlETR3 and SlETR4*, and ethylene signaling in tomato fruit (Li et al., 2016). In detached kiwifruits, exogenous application of sucrose induced an increase in the expression levels of two ethylene biosynthetic genes (Fei et al., 2020). Furthermore, *ETR* showed higher expression at the onset of tomato, grape, apple, and clementine ripening coincidently to the start of sugar accumulation, although the causal relation between both events remains to be further investigated (Chen et al., 2018). However, taken together, these findings may suggest the existence of a hub between ethylene and sucrose for fruit ripening control.

In early stages of fruit development, auxins play a key role in preventing fruit abscission, which will be induced by ethylene when the fruit is ripe (Murayama et al., 2015). In young fruits, auxins accumulate in the peduncle to avoid abscission, and thus allow sucrose transport to sink cells where it is usually degraded by CWIN. As a consequence, the resulting glucose inhibits programmed cell death, and thus promotes fruit development (Ruan, 2012). In addition, it was suggested that auxin accumulates during early stages of fruit development which promotes cell division and represses genes involved in ripening-associated processes (Medina-Puche et al., 2016). Jia et al. (2017) observed that ABA, sucrose, and auxin had an integrated regulation during grape berry ripening; indeed, apart from the synergetic action of ABA/sucrose previously commented, a negative influence of the auxin indole acetic acid on sugar accumulation was observed (**Figure 1**; Jia et al., 2017). Jia et al. (2016) showed that indole acetic acid inhibited the expression of sugar-accumulated genes during strawberry and tomato fruit ripening. Furthermore, overexpression of the auxin response factor *SlARF6* and downregulation of *SlARF4* resulted in increased soluble sugar content in tomato fruits, as a consequence of enhanced photosynthetic activity. Enhanced levels of sucrose in tomato fruits further impact starch synthesis, for which a crosstalk between auxin and sucrose metabolism at the early stages of fruit development cannot be ruled out (Sagar et al., 2013; Yuan et al., 2019).

A link between gibberellic acid (GA), fruit ripening, and sugar signaling has also been outlined, as mentioned later in this review for anthocyanin synthesis (Li et al., 2014). GA is involved in fruit development, in particular in cell division and expansion, which may depend on a complex network between GA and glucose signaling, as described in barley embryos (Perata et al., 1997). Exogenous application of GA on Cabernet Sauvignon grape cell culture was able to relieve the hexokinase-dependent repression of glucose on *CWIN* expression, suggesting a coupling between the hexose and the hormone transduction pathways to control berry development (Zhang Y. et al., 2014). In addition, transient silencing of *FaGAMYB*, a MYB TF regulated by GA, outlines the connection between GA, ABA, and sucrose pathways during Camarosa strawberry fruit ripening. Indeed, *FaGAMYB*-silenced fruits showed both sucrose and ABA decreased levels, suggesting that GA signaling may act upstream of the ABA- and sucrose-dependent ripening processes (Vallarino et al., 2015).

Sucrose and ABA treatments could also separately induce the expression of two genes involved in JA synthesis in strawberry (Jia et al., 2016). This hormone is positively affected by ABA and promotes fruit-ripening processes in a possible crosstalk with this latter and sucrose (**Figure 1**; Jia et al., 2016). However, in some climacteric fruits, JA seems to act as an inhibitor of ethylene biosynthesis pathways, thereby delaying fruit ripening, as observed in apples and pears (Kondo et al., 2000; Nham et al., 2017; Lindo-García et al., 2020). More studies are needed to fully understand the role of JA in fruit ripening and to confirm the putative existence of any crosstalk with sugars.

## Sucrose Promotes Anthocyanin and Carotenoid Synthesis During Fruit Ripening

One of the common traits of fleshy fruit ripening is the synthesis and accumulation of metabolites which function is to increase their palatability to seed dispersers (Pott et al., 2019). Some of these compounds are pigments, conferring bright and appealing colous to ripe fruits. An important group of colorful molecules synthesized during ripening are anthocyanins, which accumulate in vacuoles, conferring red, purple, pink, or blue colors to fruits such as strawberries, blueberries, eggplants, grapes, or apples (Petrussa et al., 2013). They are polyphenol molecules belonging to the flavonoid class and synthesized through the phenylpropanoid pathway, and which metabolism and regulation have been widely studied, both for their important roles *in planta* and in the human diet (Vogt, 2010; Fraser and Chapple, 2011; Hassan and Mathesius, 2012; Petrussa et al., 2013; Giampieri et al., 2015; Pott et al., 2019). In particular, their biosynthesis is mediated in higher plants through a ternary conserved complex (MBW complex), formed by R2R3-MYB, basic helix-loop-helix (bHLH) and WD40-repeat proteins, which regulates the expression of the pathway structural genes (Xie et al., 2016).

Interestingly, sucrose treatment in *Arabidopsis* was able to up-regulate several biosynthetic genes of the flavonoid/anthocyanin pathway and also to induce the expression of *MYB75/PAP1* TF, involved in this class of pigments biosynthesis. These results were concomitant with the observed increase levels of anthocyanins in mutants with enhanced content in soluble sugars and starch (Lloyd and Zakhleniuk, 2004; Teng et al., 2005; Solfanelli et al., 2006). Furthermore, the interplay between sucrose and ABA brought to light the role of sugars in anthocyanin synthesis during strawberry fruit ripening (Jia et al., 2013; Jia et al., 2016; Luo et al., 2019). However, it seems that sugar alone, without the presence of exogenous ABA is sufficient to trigger anthocyanin synthesis, as described in grape berries (Dai et al., 2014; Olivares et al., 2017). Indeed, sucrose application (90 mM applied with a hand-held sprayer) on Crimson Seedless grape berries 13 days after veraison increased anthocyanin accumulation, quickening the development of fruit skin color, where these pigments are found. Even if the effect was less accentuated than with ABA or ABA + sucrose treatments, sucrose application could be an easy handling, low-cost strategy to promote earlier harvests (Olivares et al., 2017).

**Figure 2:**
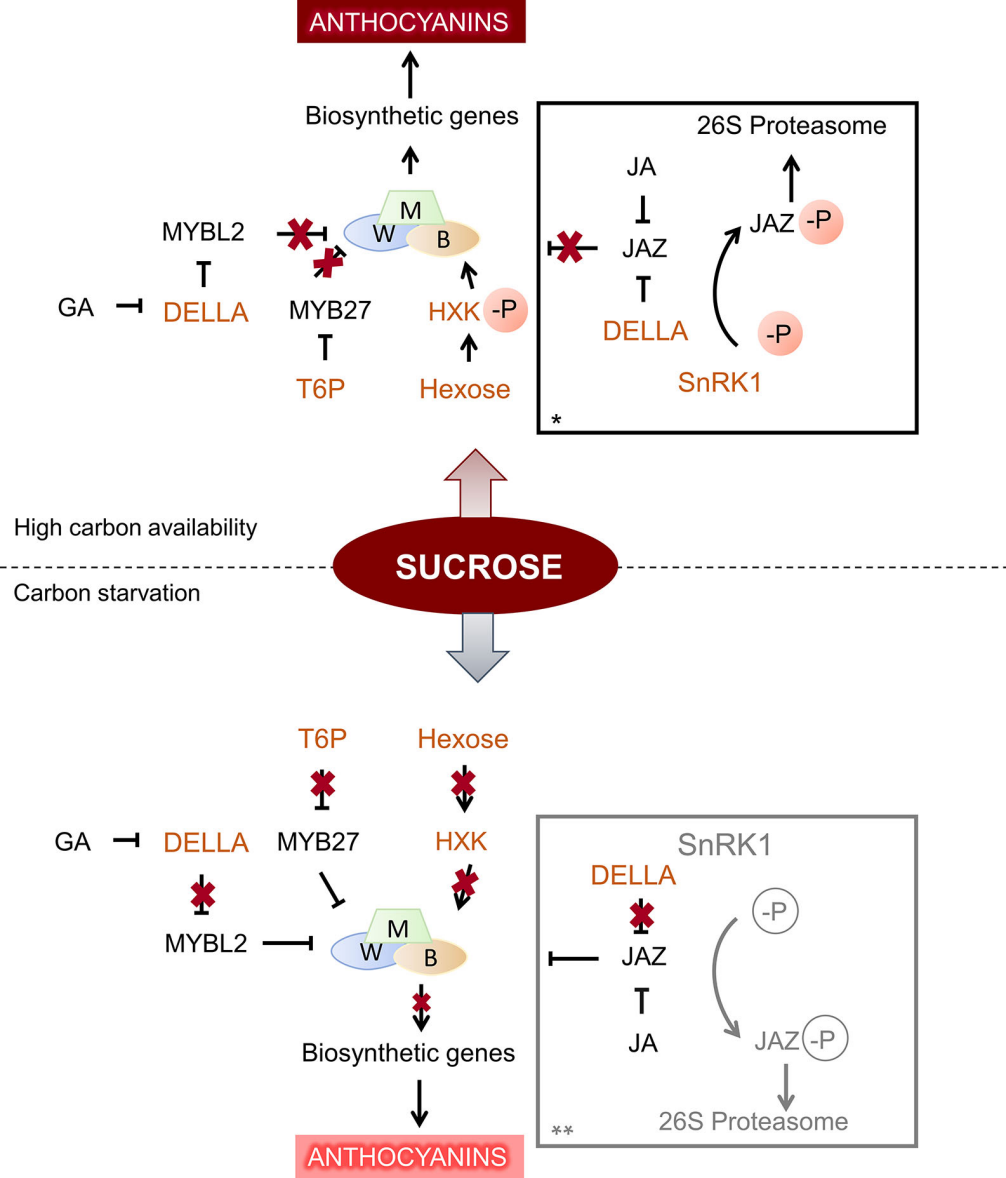
General schema of sugar signaling in anthocyanin synthesis, under high and low carbon conditions. Components of sugar signaling cascade are shown in orange. When there is a high supply of sucrose, hexokinase (HXK) is phosphorylated and activates *bHLH3* (B) transcription factor, part of the MBW complex, which promotes anthocyanin synthesis by its action upon downstream biosynthetic genes. In addition, high levels of trehalose-6-phosphate (T6P) represses *MYB27*, a general repressor of anthocyanin synthesis, leading to increased anthocyanin content showed in darker hues (Hu et al., 2016; Nardozza et al., 2020). DELLA protein degradation is also repressed under sucrose treatment, and favors anthocyanin accumulation by sequestering JAZs and MYBL2 repressors (Li et al., 2014; Qi et al., 2014; Xie et al., 2016). Under carbon starvation, HXK is no more phosphorylated, and thus does not activate *bHLH3* (B). In addition, low T6P level removes the repression upon *MYB27*, resulting in lower anthocyanin accumulation, represented in paler tone. In addition, lower DELLA stability under low sucrose and its degradation mediated by GA releases its effect upon MYBL2 and JAZ repressors (Li et al., 2014). * Under a specific range of sucrose (under sucrose oversupply, this process is inhibited, possibly by T6P repression over SnRK1), SnRK1 induces the phosphorylation of JAZ18, promoting its degradation through the 26 proteasome. JAZ18 repression upon *bHLH3* (B) is removed, allowing the MBW complex to activate downstream biosynthetic genes for anthocyanin accumulation (Liu et al., 2017). ** No data are available about SnRK1 effect under sucrose starvation (shown in gray).

Studies in grape suspension cells have allowed to understand better the mechanisms by which sugars promote anthocyanins, and to establish which are the sugars involved in anthocyanin synthesis. An early study in Gamay Fréaux cell suspension showed that sucrose (100–150 mM), glucose (150 mM), and fructose (150 mM) were able to enhance anthocyanin content, and that neither mannitol nor sorbitol induced pigment accumulation, suggesting that the sugar effect was not osmotic (Larronde et al., 1998). By applying fructose, glucose, sucrose, or different glucose analogs to sliced Cabernet Sauvignon grape disks, Zheng et al. (2009) could conclude that only sugars that can serve as substrates for hexokinase (HXK) are able to affect anthocyanin accumulation, as previously found in Gamay Fréaux cell suspension (Vitrac et al., 2000). These results suggest that HXK may act as a sensor for sugar signaling in grape cells, as suggested previously by Cho et al. (2006) in *Arabidopsis*, and consequently activate anthocyanin synthesis. HXK mechanism in modulating anthocyanin content was further studied in apple. Indeed, the activation of apple HXK1 by phosphorylation under high glucose condition allows to stabilize basic helix-loop-helix TF *MdbHLH3*, a part of the regulatory complex controlling anthocyanin synthesis at the transcription level (**Figure 2**). The HXK1-mediated anthocyanin accumulation in apple peel depends on *MdHLH3*, and was confirmed by transient expression assay in the fruit (Hu et al., 2016).

Apart from HXK role in sugar signaling for anthocyanin synthesis, the function of SnRK1 protein was put forward in *Arabidopsis* and apple calli (Baena-González et al., 2007; Liu et al., 2017). While 1% sucrose promoted anthocyanin accumulation in apple calli overexpressing *MdSnRK1.1*, higher concentration of this sugar (12%) showed inhibition. In this sense, the proposed model under low sucrose level, MdSnRK1.1 interacts with MdJAZ18, a repressor in jasmonate signaling pathway, and induces its degradation. As MdJAZ18 interacts with MdbHLH3, its degradation allows MdbHLH3 to activate anthocyanin biosynthesis (**Figure 2**; Liu et al., 2017). Furthermore, a link between JA, GA, and sucrose signaling pathways, fine-tuning anthocyanin accumulation was established in *Arabidopsis* by Li et al. (2014) and Xie et al. (2016). In particular, DELLA proteins, key GA signaling negative regulators, have been identified as components of sucrose signaling, as GA-mediated degradation of RGA, one of the five DELLA proteins found in *Arabidopsis*, was inhibited in seedlings grown with 100 mM sucrose (Li et al., 2014). It was later established that DELLA proteins, in GA absence, favored anthocyanin accumulation by sequestering the anthocyanin repressors JAZs and MYBL2, allowing the formation of an active MBW complex (Qi et al., 2014; Xie et al., 2016). While anthocyanin synthesis by the mechanisms involving JA, GA-DELLA and sugar signaling could be explained as a plant response to abiotic stresses (Tarkowski and Van den Ende, 2015; Zhang Y. et al., 2017; Wingler et al., 2020), its role in pigment accumulation during fruit ripening still remains to be determined.

Other studies outlined the effect of sugar treatment on the expression of structural genes of the flavonoid pathway. Using Gamay Red cell culture, Gollop et al. (2001, 2002) observed that the expression of *dihydroflavonol-4-reductase* and *leucoanthocyanidin dioxygenase*, two genes involved in anthocyanin pathway, were up-regulated by sucrose. Sugars were also able to increase transcript and proteins levels of flavanone 3-hydroxylase, a crucial enzyme regulating the bifurcation between the flavonol and anthocyanin branches within the flavonoid pathway (Zheng et al., 2009). In Barbera grape suspension cells, the up-regulation of different structural genes of the general phenylpropanoid pathway led to an increase in polyphenols, not restricted to the anthocyanin class, but including also other flavonoids, such as catechins, and stilbenes (Ferri et al., 2011).

Interestingly, sugar effect on pigment accumulation seems to be anthocyanin-specific, as suggested in intact Cabernet Sauvignon grape berries cultivated *in vitro* (Dai et al., 2014). Red grape berries usually accumulate five different classes of anthocyanins, cyanidin and petunidin derivatives, which confer red colors and peonidin, delphinidin, and malvidin compounds, responsible for blue hues (Liang et al., 2008). Sixty days after starting the *in vitro* culture, the delphinidin- to cyanidin-derived compounds ratio increased concomitant with sucrose, glucose, or fructose concentration. This ratio is known to depend on berry developmental stage (Falginella et al., 2012) and on the ratio of expression of two enzymes, the flavonoid 3’,5’-hydroxylase and the flavonoid 3’-hydroxylase, which drive metabolic fluxes toward delphinidin and cyanidin anthocyanins, respectively (Castellarin et al., 2006). Transcriptomic analysis pointed out that the ratio between the two enzymes, flavonoid 3’,5’-hydroxylase/flavonoid 3’-hydroxylase, was increased by higher glucose concentration in the culture medium (Dai et al., 2014). Furthermore, *in vitro* berries transcriptomic analysis highlighted that anthocyanin content enhancement by sugars results from important changes in the expression of both regulatory and structural genes of the phenylpropanoid pathways by signal reprogramming. In particular, the expression of a putative *UDP-glucose:anthocyanin 3-O-glucosyltransferase*, was up-regulated and could play a key role in anthocyanin increase, also considered a major control point in the synthesis of these compounds (Boss et al., 1996; Dai et al., 2014).

Sugar’s effect on anthocyanin regulation is not restricted to grape berries or apples. Indeed, carbon starvation in Zes006 red kiwifruit affected fruit development as well as anthocyanin concentration by affecting key structural genes of the anthocyanin pathway (Nardozza et al., 2020). Here, the authors proposed a model in which anthocyanin level modulation under carbon starvation involved trehalose-6 phosphate (**Figure 2**). Comparing anthocyanin and main sugar levels (sucrose, fructose, glucose, and sorbitol) in different varieties of apricot, a strong correlation was observed between both groups of compounds, but only in red-blushed varieties, which accumulates higher content of cyanidin derivatives (Huang et al., 2019). In Chinese bayberry fruits, a correlation between soluble sugars and anthocyanin levels were also observed in Dongkui and Biqi varieties (Shi et al., 2014). Zhao et al. (2017) identified a sucrose synthase gene, *FaSS1*, in Beinongxiang strawberry, which down-regulation led to a significant delay in fruit ripening, as well as a decrease in anthocyanin content. However, sucrose effect on anthocyanin accumulation do not appear to be universal among fruit-bearing species. Indeed, the immersion of pre- and postharvest bilberry fruits in glucose and sucrose solution (200 mM) did not induce anthocyanin biosynthesis (Karppinen et al., 2018).

As sucrose treatment has been recently proposed as a postharvest strategy to maintain fruit and vegetables quality traits, its effect (50, 270, and 500 mM) on strawberry was also evaluated (Li et al., 2019; Siebeneichler et al., 2020). While exogenous sucrose treatment increases internal glucose and fructose, the effect on pigment accumulation seems more anthocyanin-specific. Indeed, from the two main anthocyanin classes found in strawberry, only pelargonidin-derived compounds were increased, while no changes were observed in cyanidin-derived pigments (Li et al., 2019). This differential effect on anthocyanin content could be explained by the fact that exogenous sucrose application up-regulated the expression of all structural genes of the flavonoid pathway, with the exception of the *flavonoid 3’-hydroxylase*, which, as mentioned above, drive the metabolic flux to the synthesis of cyanidin-derived anthocyanins (Castellarin et al., 2006). However, the exact role of sucrose treatment in anthocyanin accumulation during fruit postharvest remains to be determined, as a function as mere metabolic substrate cannot be ruled out (Siebeneichler et al., 2020).

**Figure 3:**
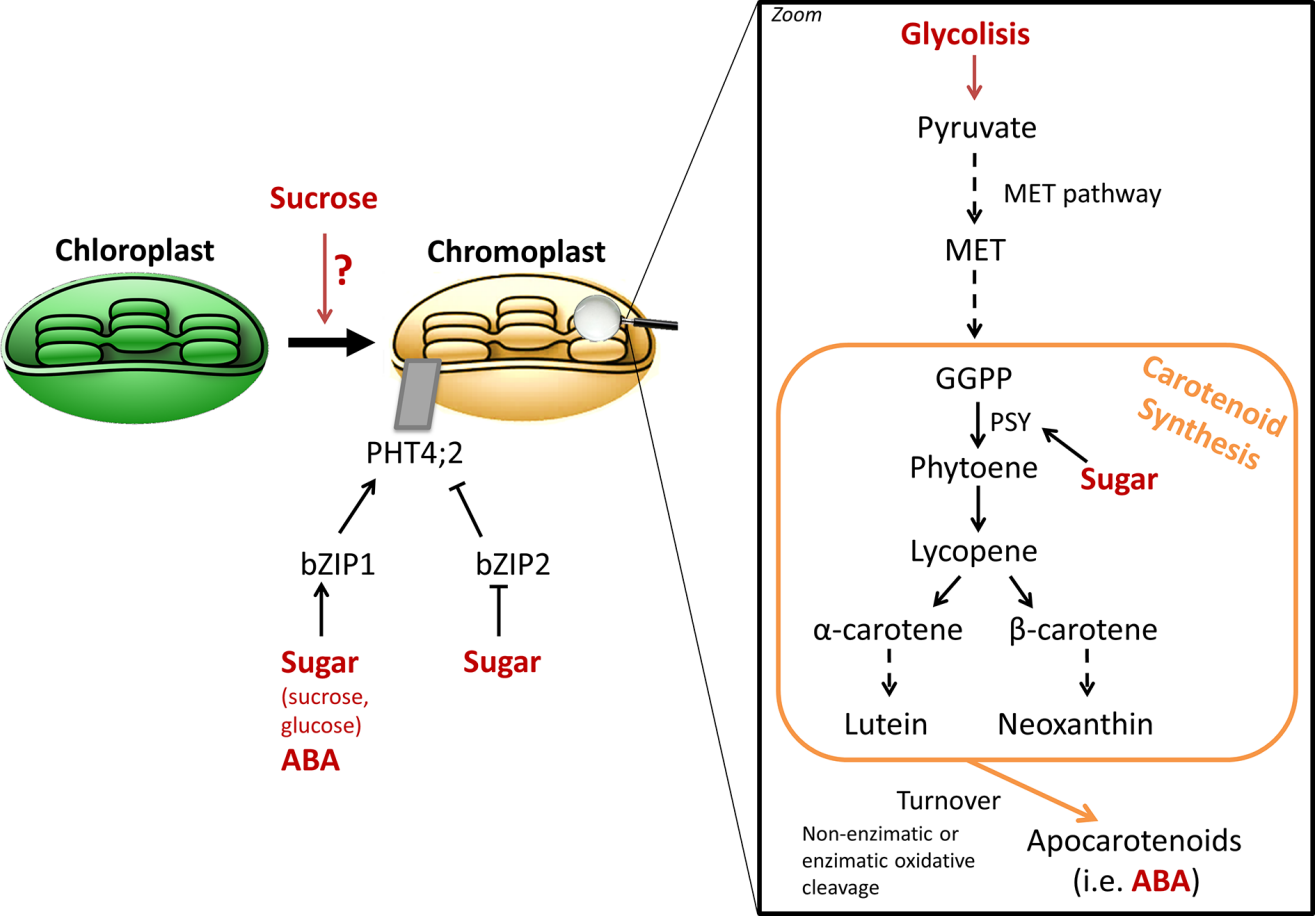
General schema of sugar signaling in carotenoid synthesis. Several evidences highlight the relationship between sugar content and carotenogenesis (Zhang et al., 2017; Heng et al., 2019). While sucrose appears to be involved chromoplast differentiation (Iglesias et al., 2001; Fanciullino et al., 2014), the molecular cascade remains unknown. Modulation of *ClPHT4;2* expression through bZIP TF, which in turn are regulated by sugars and ABA, has been described to be involved in carotenoid accumulation during domesticated watermelon ripening (Zhang et al., 2017). While sugar catabolism can fuel carotenoid biosynthetic pathway by providing precursor building blocks through glycolysis, a regulating role over the pathway seems to exist, as demonstrated by the effect upon *phytoene synthase* (PSY) expression (Télef et al., 2006). In addition, sugar effect on carotenoid synthesis may also impact its crosstalk with ABA, as carotenoid turnover yields apocarotenoids, which serve as ABA precursors.

Carotenoids are another class of important pigments, which accumulate during fruit ripening and are responsible for the bright yellow and red tones of many ripe fruits, including tomato, watermelon or citrus (Sun et al., 2018). Carotenoids are tetraterpenoid compounds, which synthesis takes place through the methyleryhtritol 4-phosphate pathway (Eisenreich et al., 2004). Interestingly, they also provide precursors for ABA synthesis, and are essential components of the human diet (Sun et al., 2018). A first study in Okitsu mandarin outlined that sucrose supplementation in the peel accelerated the conversion of chloroplasts to chromoplasts, which characteristic is the massive accumulation of carotenoids, during citrus ripening, and thus promoting color break (Iglesias et al., 2001). Addition of ethylene, known to mediate chlorophyll degradation and thus color change, to sucrose-treated mandarin epicarps had no additive effect, therefore, it was suggested that both signaling molecules are components of the same pathway or share common transductor elements (Iglesias et al., 2001). The effect of exogenous sucrose on carotenoid accumulation was evaluated using mature green Aisla Craig tomato pericarp discs (Télef et al., 2006). Only lycopene and phytoene were enhanced under sucrose treatment, while no significant differences were observed in other carotenoids. However, it was also established that sucrose is not vital to induce carotenoid synthesis and requires ethylene signaling (Télef et al., 2006). Furthermore, upregulation of *phytoene synthase* (*PSY*) gene by sucrose, the enzyme catalyzing the first committed step of carotenoid synthesis, has been proposed to positively affect ABA levels, concomitant with the evidences discussed in the previous section (**Figure 3**; Falchi et al., 2013). PSY catalyzed the first committed step of carotenoid synthesis and is considered a major control point of the pathway. Télef et al. (2006) demonstrated that sugar treatment in tomato pericarp discs accelerated the accumulation of *PSY1* mRNA, while no effect was observed on other enzymes of the pathway. Mandarin, orange, and lemon juice sacs cultured *in vitro* also allowed to observe the promotion of carotenoid accumulation by sucrose treatment, and *PSY* upregulation (Zhang et al., 2012). However, the molecular mechanism by which sucrose regulates carotenoid metabolism at the transcriptional level is still unknown.

A recent study in watermelon established a possible molecular connection between sugar (glucose) signaling and carotenoid accumulation (Zhang J. et al., 2017). Indeed, a phosphate transporter, ClPHT4;2 was identified in chromoplast membranes, and which expression during fruit ripening was associated with carotenoid accumulation in the fruit flesh. *ClPHT4;2* expression is modulated by two bZIP TF, ClbZIP1 and ClbZIP2, which in turn are regulated both by sugar and ABA signaling pathways (**Figure 3**; Zhang J. et al., 2017). In fact, the authors of the study proposed a model in which ABA and sugars increase *ClPHT4;2* expression by the binding of ClbZIP1 TF to motifs “ABA and osmotic stress response elements” present in the promotor sequence. On the opposite, the absence of these *cis*-elements in wild watermelon lines would not modulate *ClPHT4;2* expression, which remains low along ripening, and may explain the pale color of the non-domesticated watermelon accessions. This association between sugars, carotenoid, and *ClPHT4;2* gene expression is of particular interest, as it outlined a regulatory relation between carbohydrates and pigments during the evolution of domesticated watermelon from uncolored and non-sweet wild progenitors (Zhang J. et al., 2017; Ren et al., 2018).

A combination of proteomic and metabolomic approaches outlined that carotenogenesis ***in planta*** in banana pulp is associated with enhanced soluble sugar content and carbohydrate metabolism (Heng et al., 2019). On one hand, it cannot be ruled out that pigment accumulation is favored by sucrose degradation and a higher glycolytic activity to fuel the carotenoid biosynthetic pathway, as it has been observed in transgenic maize and golden rice (**Figure 3**; Decourcelle et al., 2015; Gayen et al., 2016). On the other hand, a plausible role for sugar signaling in plastid development and the integration of carbon and redox status, which in turn depends strongly on sugar metabolism, to regulate carotenoid synthesis was suggested by Fanciullino et al. (2014). However, the components of the signaling cascades linking sugars to plastid formation and carotenoid accumulation are still missing (Fanciullino et al., 2014; Heng et al., 2019).

## Sugar Signaling Is Involved in Abiotic and Biotic Stress Responses During Fruit Development

During the last decades, consequences of global warming, *i.e.* increased abiotic stresses on plants, are challenging crop productivity (Rampino et al., 2012). In this sense, fruit and seed development involves key processes for global production, and those have been described to be particularly sensitive to heat or drought stresses. It known that abiotic stress during early reproductive organ formation leads to abnormal growth and fruit abortion (Thakur et al., 2010). Several evidence outlined the role of sugars as signaling molecules in abiotic stress responses in plant (Ruan et al., 2010; Ruan, 2012; Ruan, 2014). The little information available to date for sugar transduction pathways in responses to abiotic stresses during fruit development and ripening is reviewed in the next paragraphs.

Sucrose import to the fruit, together with invertase activity are especially susceptible to abiotic stresses; CWIN and VIN activities have been linked to ovary abortion under drought in maize (McLaughlin and Boyer, 2004). In Moneymaker tomato, RNAi-mediated silencing a flower and fruit specific CWIN gene, *LIN5*, had negative consequences on the reproductive organs, by increasing fruit abortion, reducing fruit size and seed number per plant (Zanor et al., 2009). While the higher rate of fruit abortion has been directly correlated with low sucrose content in the ovary tissue in many species (McLaughlin and Boyer, 2004), the aberrant fruit phenotypes are probably the result of altered hormonal balance in the silenced tomato lines, as a consequence of the crosstalk between sugars and hormones (Zanor et al., 2009). On the opposite, silencing the expression of its inhibitor (*INVINH1*) leads to an enhanced CWIN activity, which in turn positively impacts fruit and seed development (Jin et al., 2009). Furthermore, increased CWIN activity through *INVINH1* silencing enhanced sucrose import and allowed to sustain tomato fruit set under long-term moderate heat stress (28/20°C, day/night) (Liu et al., 2016). CWIN protective effect was most likely achieved by providing energy for the synthesis of heat stress TFs and heat shock proteins, which are key mechanisms for heat tolerance, or by favoring auxin synthesis, a key hormone impacting fruit set. In addition, Liu et al. (2016) also corroborated that increased CWIN activity results in an inhibition of programmed cell death both under long-term moderate and short-term severe heat stresses, in an oxidative independent- and dependent-response, respectively. Interestingly, a recent study in XF-2 tomato plants suggests that high CWIN activity is also requires for cold tolerance. Indeed, *INVINH1* silencing results in enhanced chilling resistance (Xu et al., 2017).

By comparing CWIN, VIN, sugar import and sucrose synthase activities in heat-tolerant and heat-sensitive tomato lines under optimal (25°/20° day/night) and heat-stress conditions (5° above normal), Li et al. (2012) showed that not only CWIN (LIN7) but also VIN may be involved in heat tolerance in tomato reproductive organs, as their activities were higher in the former genotype young fruits. In addition, heat-tolerant plants showed a higher fruit to vegetative biomass ratio than susceptible lines, without significant changes in photosynthesis efficiency, suggesting that the former lines have a higher phloem import rate. Finally, and in agreement with the results obtained by CWIN overexpression (Liu et al., 2016), the expression of a *PLDa1*, a programmed cell death marker was found to be higher in the heat-sensible tomato plants, and could be related to invertase activities. Indeed, based on studies in maize (McLaughlin and Boyer, 2004; Boyer and McLaughlin, 2007), it is reasonable to hypothesize that the higher amounts of hexoses generated by invertase activity in heat-tolerant genotype can suppress the expression of *PLDa1*, and probably other programmed cell death-related genes (Li et al., 2012). Taken together, the results discussed here seem to confirm the role of invertases in connecting sugar signaling and fruit heat tolerance.

In addition, impairment of sugar metabolism in tomato is also observed under high salinity, resulting in decreased sink strength and activity, and thus fruit yield (Albacete et al., 2014). Heterologous overexpression of *CIN1*, a cell wall invertase gene, under the control of a putative fruit promotor was able to decrease the negative impact of salinity on yield, showing also altered hormonal levels (*i.e.* increased content of indole acetic acid [an auxin], *trans-*zeatin [a cytokinin], and ABA and decreased concentration of 1-aminocyclopropane-1-carboxylic acid, the precursor of ethylene). The interplay between CIN1 and hormones remains still to be further clarify, but it appears that it promotes fruit set by inducing cell division and expansion, and adjusts sink strength to sugar availability (Albacete et al., 2014).

Apart from the role of sucrose and hexoses described in heat stress responses, accumulation of the sugar alcohol galactinol was observed in Cabernet Sauvignon grape berries under heat stress (Pillet et al., 2012). As no induction of the raffinose family oligosaccharide pathway was observed, in which galactinol production is the first committed step, it was suggested that galactinol could act as a signal metabolite under grape heat stress, in order to trigger adaptive responses (Pillet et al., 2012).

Other hints towards the role of sugar signaling in abiotic or biotic fruit stress tolerance were found in the possible role of some SWEET transporters (Chen et al., 2010; Chong et al., 2014; Miao et al., 2017; Lu et al., 2019). The heterologous expression of apple *MdSWEET17* in Tianjinbaiguo tomato induced fructose accumulation in the fruits. Additionally, the resistance of the transgenic plants to drought was increased, suggesting that fructose signaling may be involved in stress tolerance (Lu et al., 2019). Interaction and co-expression network analysis outlined the strong transcriptional response of some SWEET transporters in early Baxi Jiao banana fruit development and under abiotic/biotic stresses, suggesting an enhanced sugar transport under environmental pressure (Miao et al., 2017).

Sugar signaling seems to be also affected under biotic stress, as described in grape berries (Vega et al., 2011; Chong et al., 2014). Glucose transporter *VvSWEET4* is strongly induced in response to the necrotrophic fungus *Botrytris cinerea* (Chong et al., 2014). While a possible explanation of this up-regulation would be the manipulation of the host resources, *i.e.* sugars, by the pathogen, an alternative hypothesis would be the possible function of VvSWEET4 in plant cell death. Indeed, *Arabidopsis* mutants for *SWEET4* were found to be more resistant to *B. cinerea* and to show reduced cell death, which is required by the fungus to infect the host (van Kan, 2006). In Cabernet Sauvignon berries infected with Grapevine leaf-roll-associated virus-3, the expression of genes implicated in sugar metabolism and transport was decreased, concomitant with reduced accumulation of fructose and glucose. Possibly as a consequence of sugar content decrease, the infection by the virus also induced a reduction in anthocyanin content and in the transcript levels of several structural and regulatory genes of the anthocyanin pathway (Vega et al., 2011).

## Conclusion

Carbohydrates are the main components of plant tissues, being needed both for their structural role and energy obtaining. However, very little is still known about the transduction pathways which convert them into signaling molecules, allowing the plant to monitor sugar status to optimize growth and development, including fruit set and ripening. Here we reviewed the role of sugars, alone or in a crosstalk with hormone signaling pathway, in controlling important events in fruit ripening, such as the accumulation of health-promoting pigments. Furthermore, we highlighted the need for a deeper understanding of the regulatory mechanisms underlying sugar and hormone connection in controlling fruit set, growth, and ripening, as this will permit to have a better control on crop yield and productivity, an urgent issue due to overpopulation and global warming.

## Author Contributions

All authors contributed to the article and approved the submitted version.

## Funding

This work was supported through grants RTI2018-099797-B-100 (Ministerio de Ciencia, Innovación y Universidades, Spain) and UMA18-FEDERJA-179 (FEDER-Junta Andalucía). In addition, we acknowledge partial funding by the European Union’s H2020 Programme (GoodBerry; grant number 679303). SD-S and SO acknowledge the support by Plan Propio from University of Malaga.

## Conflict of Interest

The authors declare that the submitted work was carried out in the absence of any commercial or financial relationships that could be construed as a potential conflict of interest.
